# The Effect of Vascular Endothelial Growth Factor on Bone Marrow Mesenchymal Stem Cell Engraftment in Rat Fibrotic Liver upon Transplantation

**DOI:** 10.1155/2019/5310202

**Published:** 2019-12-04

**Authors:** Ke Yuan, Chunyou Lai, Lingling Wei, Tianhang Feng, Qinyan Yang, Tianying Zhang, Tao Lan, Yutong Yao, Guangming Xiang, Xiaolun Huang

**Affiliations:** ^1^Department of Hepatobiliary-Pancreatic Surgery Center and Cell Transplantation Center, Sichuan Academy of Medical Sciences & Sichuan Provincial People's Hospital, Sichuan, China; ^2^School of Medicine, University of Electronic Science and Technology of China, Chengdu, Sichuan, China

## Abstract

**Background:**

According to existing related experiments and research reports, stem cell transplantation therapy has been shown to have a positive effect on the recovery of liver fibrosis/cirrhosis, but for some reason, this therapy still cannot be widely used in clinical work. One of the reasons that cannot be ignored is the low quantity of exogenous stem cells transplanted into the liver in vivo. Thus, we investigated whether the use of the vascular endothelial growth factor (VEGF) can increase the number of stem cell transplants and improve the efficacy of stem cell transplantation therapy.

**Methods:**

Using a Sprague-Dawley rat liver fibrosis model, we transplanted into fibrosis liver allograft bone marrow mesenchymal stem cells (BMSCs) which were labelled with chlormethylbenzamido-1,1-dioctadecyl-3,3,3′3′-tetramethylin-docarbocyamine (CM-DiI) or injected VEGF adenovirus solution through the tail vein or conducted the above two operations simultaneously. The cell surface receptor profile of BMSC was examined by flow cytometry and immunofluorescence staining. Hepatic sinusoidal vascular leakage was measured with Evan's blue dye assay. Paraffin section staining, immunofluorescent staining, RT-qPCR (quantitative reverse transcription polymerase chain reaction), and Western blot were used to evaluate hepatic pathological changes and physiology function.

**Result:**

The in vivo study indicated that, comparing with other groups of rats, the rats with combined treatment of BMSC transplantation and VEGF injection exhibited obvious reduction in liver fibrosis. Evan's blue dye assay suggests that after injecting with VEGF adenovirus solution, the rat's hepatic sinusoidal permeability would be increased. We confirmed the expression of very late antigen-4 (VLA4, integrin *α*_4_*β*_1_) on rat BMSCs and the elevated expression of vascular adhesion molecule-1 (VCAM-1) in the hepatic sinusoidal endothelial cells. In addition, the analysis of CM-DiI-labeled BMSCs showed that the BMSC+VEGF group exhibited better cell engraftment and that the engrafted cells were mainly distributed in the hepatic parenchyma. Furthermore, compared with the other situation, it is best to reconstitute the liver secretion and regeneration function of rats after combined application of VEGF and BMSC.

**Conclusion:**

We showed that VEGF promotes the engraftment of BMSCs in liver fibrosis, enhances liver regeneration, and improves liver function. These outcomes may be related to the increasing hepatic sinusoidal endothelium permeability and VCAM-1-increased expression.

## 1. Background

Liver fibrosis is a chronic disease resulting from repeated injuries to the liver, caused by infection, drug toxicity, metabolic disorders, and immune cytotoxicity [1]. Fibrosis is characterized by the unique septa composed of extracellular matrix proteins and forms through the interaction of multiple cells and factors [2, 3]. Clinically, the end-stage liver fibrosis is termed cirrhosis denoting significant impairment in liver function. Liver fibrosis/cirrhosis is the 13^th^ leading cause of death in adults worldwide (8^th^ in the U.S.) and results in approximately 103 million deaths each year [4, 5]. Treatments for liver fibrosis/cirrhosis are limited, and liver transplant is the most well-known therapeutic strategy. However, due to the scarcity of available donor livers, not every patient in need of liver transplant can be treated. Specifically, studies have shown that only one-third of patients with end-stage liver disease were able to receive liver transplant, and in the next 20 years, the demand for liver transplant will increase by 23% [6, 7]. Therefore, there is an urgent need to develop alternative treatments for patients with chronic liver diseases. Importantly, application of stem cell therapy in the treatment of liver fibrosis/cirrhosis has become a hot topic in recent years. Particularly, mesenchymal stem cells (MSCs) differentiate into liver-like cells after engrafting into the liver, facilitate liver regeneration, and regulate immune system function to reduce liver damage [8–10]. However, one of the key challenges linked with application of MSCs as a treatment for liver fibrosis/cirrhosis is the small number of engrafted MSCs in the liver which may limit their beneficial functions [11, 12].

VEGF increases vascular permeability and was firstly reported by Senger et al. [13]. The hepatic sinusoidal endothelium is fenestrated and composed of unique endothelial cells with specific channels (pore size 6–9 *μ*m), which lack basal membrane and exhibit high permeability [12, 14]. VEGF increases the pore size and permeability of hepatic sinusoidal endothelial cells, which facilitates the recruitment of circulating cells (such as monocytes) to the liver parenchyma through hepatic sinusoidal endothelium [15, 16]. Furthermore, VEGF promotes the expression of certain stem cell-specific receptors on endothelial cells, such as VCAM-1. VCAM-1 specifically binds to stem cell surface receptor *α*_4_*β*_1_ integrin and mediates the migration of exogenous stem cells to the liver parenchyma [17, 18]. Based on the available literature, we therefore hypothesize that VEGF facilitates exogenous stem cell engraftment by increasing the permeability of the hepatic sinusoidal endothelium and inducing VCAM-1 expression. In this study, we investigated the effect of VEGF on BMSC engraftment in the liver by using a fibrotic liver rat model.

## 2. Materials and Methods

### 2.1. Experimental Animals

Mature female Sprague-Dawley (SD) rats (8-week-old, 180–220 g) and immature SD female rats (2-week-old) were purchased from the Institute of Laboratory Animals of Sichuan Academy of Medical Sciences & Sichuan Provincial People's Hospital (Chengdu, Sichuan, China). Mature rats were kept in sterile cages, with a temperature of 23 ± 1°C and a relative humidity of 45 ± 5%. All animal experiments were reviewed and approved by the Institutional Animal Experimental Ethics Committee of the Institute of Laboratory Animals of Sichuan Academy of Medical Sciences & Sichuan Provincial People's Hospital (Chengdu, Sichuan, China; approval). The procedures were performed in accordance with the Regulations of the Administration of Affairs Concerning Experimental Animals (China, 1988).

### 2.2. Liver Fibrosis Rat Model

Following one week of acclimation, 8-week-old rats were administered with 40% carbon tetrachloride (CCl_4_, Hengxing, Tianjin, China) under sterile conditions at 0.3 mL/100 g of body weight via intraperitoneal injection, twice per week for 12 weeks. After 12 weeks of injections, 6 rats were randomly selected from surviving rats and euthanized. The caudate lobe was taken for tissue sectioning and staining to make sure the fibrosis level was uniform in those rats. Masson staining was used to examine the presence of collagen to confirm liver fibrosis.

### 2.3. BMSC Isolation, Culturing, and Identification

The isolation and culture of BMSC were conducted according to previously published methods [19]. In brief, bone marrow cells were collected from the femurs and tibias of the 2-week-old SD rats. The bone marrow cells were collected into a single cell suspension and spun at 800 × *g* for 5 mins, the supernatant was removed, and complete culture medium (CCM) was added to resuspend the cells. BMSCs were cultured in 25 cm^2^ culture flasks at a density of 1 × 10^9^ cells/L. Cells were maintained at 37°C, 5% CO_2_ in a saturated humidity incubator (DHP-9082, Yiheng Company, Shanghai, China). The CCM comprised of DMEM-Low Glucose (SH30021.01B, HyClone, Logan, Utah, USA) containing 10% fetal bovine serum (FBS; 10099, Gibco, Carlsbad, CA, USA) and 0.5% penicillin/streptomycin (ST488, Beyotime, Haimen, Jiangsu, China). BMSCs were purified from bone marrow cells using the adherent method. Once the cells reached 70–80% confluence, they were harvested for successive passages. The third passage of BMSCs at the logarithmic phase was collected for transplantation.

### 2.4. Flow Cytometry

The P3 rat BMSCs were washed with PBS twice and dissociated with 0.25% trypsin-EDTA (25200, Gibco). The BMSC suspension was then centrifuged at 250 × *g* for 5 min at 27°C to collect cell sedimentation. The BMSCs were washed twice again with PBS and blocked with 5% normal goat serum for 1 h at 4°C. Then, the BMSCs were incubated with fluorescein-labeled antibodies, including anti-CD90 (1 : 100, ab225, Abcam, San Francisco, CA, U.S.), anti-CD29 (1 : 100, ab78502, Abcam), anti-CD45 (1 : 100, ab30446, Abcam), and anti-VLA4 (1 : 100, ab25247, Abcam) antibodies. The nonspecific rabbit IgG served as an isotype control. Afterward, fluorescence signals of BMSCs were analyzed quantitatively through a BriCyte E6 (Mindray DS US Inc., NJ, USA) flow cytometer at a wavelength of 488 nm and FlowJo 7.6.1 software (Tree Star, Inc., Ashland, OR, USA).

### 2.5. In Vivo Treatment Groups

The fibrosis model SD rats were randomly divided into 4 groups (*n* = 6 rats/group), including the model group, the VEGF group, the BMSC group, and the BMSC+VEGF group. Starting from the first week, the VEGF group and the BMSC+VEGF group were intravenously injected once with VEGF-overexpressing adenovirus (AdVEGF, OBiO Technology, Shanghai, China) at 3 × 10^9^ ifu (0.5 mL), once a week for 4 weeks. The other two groups were intravenously injected with saline (0.5 mL), once a week for 4 weeks. At the second week, rats in all groups were anesthetized with 3% pentobarbital (3 mg/100 g, MFCD00070198, Sigma, St. Louis, MO, USA) prior to laparotomy. For the BMSC+VEGF group and the BMSC group, 1.0 × 10^6^ BMSC suspension (0.5 mL) was transplanted via injection into the portal vein using a BD insulin syringe (Becton, Dickinson and Company, Shanghai, China). For the other two groups, 0.5 mL saline was injected via the hepatic portal vein. In each group, the rats were sacrificed 28 days after BMSC transplantation. The papillary lobe of all animals was taken for biopsy during surgery in order to ensure the same liver fibrosis in each group. Moreover, in order to determine whether adenovirus plays a role in liver fibrosis, we established two new groups (*n* = 3); liver fibrosis rats were treated with BMSCs alone and with BMSC+adenoviral vector. Starting from the first week, the BMSC+adenoviral vector group was intravenously injected with adenovirus vector (OBiO Technology, Shanghai, China) at 3 × 10^9^ ifu (0.5 mL), once a week for 4 weeks, and the BMSC alone group was intravenously injected with saline (0.5 mL), once a week for 4 weeks. At the second week, 1.0 × 10^6^ BMSC suspension (0.5 mL) was transplanted via injection into the portal vein using a BD insulin syringe. Rats of those groups were also sacrificed 28 days after BMSC transplantation, and papillary lobe samples were taken for examining by pathological section. All the operations were performed in a sterile environment, and the animals were brought back to their cages after waking up from anesthesia.

### 2.6. Preparation of Liver Histology Specimens

#### 2.6.1. Paraffin-Embedded Liver Tissue Sections

Fresh liver tissue was fixed in 4% paraformaldehyde (AR1068, BosterBio, Pleasanton, CA, USA) for at least 24 h. After dehydration in gradient alcohols, the tissue was embedded in paraffin (Taikang, TKY-BMB, Hubei, China) and sectioned using a freezing microtome (SLEE, MNT, Mainz, Germany) at the thickness of 4 *μ*m.

#### 2.6.2. Hematoxylin-Eosin Staining (H&E Staining)

The staining was performed according to the protocol published by Cardiff et al. [20]. Briefly, paraffin-embedded liver sections were dewaxed, rehydrated, and stained with hematoxylin and eosin (C0105, Solarbio Science & Technology, Beijing, China) to visualize nuclei and cytoplasm. Next, the sections were dehydrated and cleared in xylene, and coverslips were mounted with synthetic media (Permount, Zhanyun, Shanghai, China). The sections were analyzed under a biological microscope (Olympus, CX43, Shinjuku-ku, Tokyo, Japan) and images were taken.

#### 2.6.3. Masson Staining

Paraffin-embedded liver sections were dewaxed, rehydrated, and stained with hematoxylin and Ponceau S followed by phosphomolybdic acid treatment and aniline blue staining (415049, Sigma, St. Louis, MO, USA). The slides were differentiated in 1% acetic acid for 1 min. The sections were examined using a microscope (CX43, Olympus) and images were captured.

#### 2.6.4. Sirius Red Staining

Paraffin-embedded liver sections were dewaxed, rehydrated, stained in saturated Picro-Sirius red solution (365548, Sigma) for 8 min, washed with 100% ethanol, dried in an oven at 60°C, cleared in xylene for 5 min, and mounted in a neutral balsam mounting medium (E675007, Solarbio Science & Technology, Beijing, China). The sections were examined using a microscope (CX43, Olympus), and representative images were captured.

### 2.7. Immunofluorescent (IF) Staining

Immunofluorescent staining of BMSC surface markers was performed in six-hole plates. The cells in the six-hole plates were cultured in an incubator (Thermo, Waltham, MA, U.S.) at 37°C, 5% CO_2_, and saturated humidity. The cells were grown and fused in a cover slide to 95%-100%, fixed with 4% formaldehyde at room temperature for 30 min after washing 3 times, rinsed with 0.2%Triton X-100 (Bomei Biotechnology Co., Hefei, China) for 3 min, and then blocked in serum for 30 min in room temperature, followed by primary antibody incubation overnight at 4°C: anti-CD29 antibody (1 *μ*g/mL, ab95623, Abcam, San Francisco, U.S.), anti-CD45 antibody (1 *μ*g/mL, ab25386, Abcam, San Francisco, U.S.), and anti-CD34 antibody (1 *μ*g/mL, ab81289, Abcam, San Francisco.). After washing 3 times, a secondary antibody (1 : 1000, goat anti-mouse IgM mu chain, ab97228, Abcam, San Francisco, U.S.) was used to incubated for 30 min at room temperature in the dark. Sections were washed again and sealed with 95% glycerin. Paraffin-embedded liver sections were prepared as described above. Paraffin-embedded liver sections were dewaxed and rehydrated. Antigen retrieval was performed using EDTA citric acid (pH 6.0) antigen retrieval buffer (G1202, Servicebio, Wuhan, Hubei, China) in an 80°C thermostatic water bath (Jinpai, HH-600, Shanghai, China). After the slides cooled down, they were washed 3 times (5 min each) on a shaker. The sections were circled using a histology pen and quenched with an autofluorescence quencher (G1221, Servicebio) for 5 min. Following a wash step (10 min), sections were blocked in serum for 30 min, followed by primary antibody incubation overnight at 4°C: anti-*α*-SMA (*α*-smooth muscle actin, 1 : 500, GB13044, Servicebio), anti-collagen III (1 : 200, ab7778, Abcam), anti-VEGF (1 : 200, ab39250, Abcam), anti-VCAM-1 (1 : 100, ab134047, Abcam), anti-PCNA (proliferating cell nuclear antigen, 1 : 1000, GB11010, Servicebio), and anti-Ki67 (1 : 1000, ab15580, Abcam). Sections were washed 3 times on a shaker (5 min each), followed by a secondary antibody (goat anti-rabbit Alexa-488, ab150077, 1 : 500, Abcam) incubation for 50 min at room temperature in the dark. Sections were washed 3 times, followed by DAPI nuclear staining (G1012, Servicebio). Sections were washed again and mounted with antifade mounting medium (G1401, Servicebio). The slides were analyzed under a fluorescent microscope and images were captured.

### 2.8. Western Blot

Total protein samples were extracted from liver tissue using RIPA buffer (G2002, Servicebio), and the protein concentration was determined using a BCA protein assay kit (G2026, Servicebio). Protein samples were resolved using 10% SDS-PAGE and then transferred onto a PVDF membrane (IPVH00010, Millipore, Danvers, MA, USA). Membranes were blocked and incubated with primary antibodies overnight at 4°C. A total of 8 commercial antibodies were used for Western blotting, including anti-*α*-SMA (1 : 1000, Boster, USA), anti-TGF*β*1 (transforming growth factor beta, 1 : 1000, Abcam), anti-collagen III (1 : 1000, ab6310, Abcam), anti-VEGF (1 : 1000, ab46154, Abcam), anti-CYP3A1 (1 : 1000, ab22724, Abcam), anti-ALB (albumin, 1 : 1000, Abcam), anti-VCAM-1 (1 : 1000, Abcam), and *β*-actin (1 : 1000, Servicebio). Membranes were incubated at room temperature with an appropriate secondary antibody, and proteins were detected using an enhanced chemiluminescence method. Protein expression was normalized to the expression of *β*-actin which was used as an internal control.

### 2.9. RT-qPCR

Total RNA was extracted from liver tissues using a TRIzol reagent (G3013, Servicebio). Next, cDNA was synthesized using a RevertAid M-MuLV reverse transcriptase (RevertAid First Strand cDNA Synthesis Kit, K1622, Thermo Scientific, Waltham, MA, USA) with 2 *μ*g total RNA and oligo dT18-primers. RT-qPCR was performed in triplicate, and each 25 *μ*L reaction consisted of 2x qPCR mix (12.5 *μ*L), 7.5 *μ*M gene-specific primers (2.0 *μ*L), cDNA template (2.5 *μ*L), and ddH_2_O (8.0 *μ*L). Primer sequences are listed in Table 1. Gene expression results were normalized to the endogenous GADPH mRNA expression. Thermocycler conditions were as follows: an initial hold at 95°C for 10 min, followed by 40 cycles of a two-step PCR program of 95°C for 15 sec and 60°C for 60 sec on an anABI7500 system (ABI, Foster City, CA, USA). Data were collected from the same instrument and quantitatively analyzed. The expression level of each target mRNA was expressed as a fold change relative to the untreated control. Data was analyzed using GraphPad software version 6.

### 2.10. Fluorescent Labeling of BMSCs

BMSCs at the second passage (1 × 10^6^ cells) were resuspended in PBS (1 mL) and mixed with 5 *μ*L of 1 g/L fluorescent dye (CM-DiI, 40718ES50, Yeasen, Shanghai, China) for 5 min at 37°C and then moved to 4°C for 15 min. The final concentration of CM-DiI was 5 *μ*mol/L. Finally, the cells were washed with PBS to remove the unbound CM-DiI.

### 2.11. Measurement of Hepatic Sinusoidal Endothelium Permeability

Evan's blue (Sigma) staining was used to evaluate the permeability of sinusoidal endothelium to albumin. This technique is based on the principle that Evan's blue dye binds avidly to the negatively charged intravascular albumin and is therefore a reliable estimate of transvascular fluxes of macromolecules. 28 days after BMSC transplantation, rats were anesthetized with 3% pentobarbital (3 mg/100 g) and then injected with 2% Evan's blue dye (30 mg/kg) via the femoral vein. The dye was allowed to circulate in the rats for 30 minutes before the livers were perfused with heparin- (50 U/mL) containing PBS. Perfusion was stopped when the effluent became clear, and the livers were harvested. The livers were then dried for 48 h at 60°C and weighed. The entire liver was homogenized in PBS (0.1 mg tissue/1 mL) and incubated with double the volume of formamide for 18 h at 56°C. The tissue homogenate was then spun at 5000 × *g* for 30 min. Next, 1 mL of the supernatant was collected, and the amount of Evan's blue was examined using a spectrophotometer (SP-756P, Spectrum, Shanghai, China) at 620 nm. The results were expressed as the amount of Evan's blue per gram of tissue, where the higher amount of Evan's blue dye captured from tissue (per gram) was linked with the higher vessel permeability in the tissue [18, 19].

### 2.12. Liver Fibrosis Scoring Criteria

In order to allow readers to have a direct and accurate understanding of the pathological changes in the liver of each group of rats in our experiment, we took the method of double blindness for research with the Laennec fibrosis scoring system [21] to assess liver fibrosis.  Table 2 has the specific details of the score criteria.

### 2.13. Statistics

Unpaired Student's *t*-test was used to make comparisons between two experimental groups. When three or more groups were compared, multiple comparisons were made using ANOVA followed by the Bonferroni post hoc test to identify significance. Values were considered significant for *p* < 0.05.

## 3. Results

**Figure 1:**
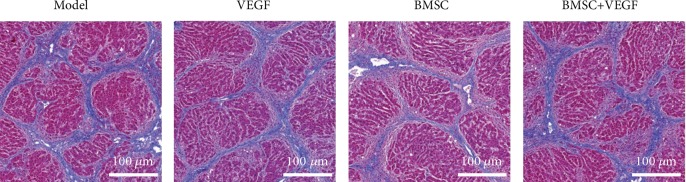
Masson staining of the papillary process in four groups of liver fibrosis model rats. The blue stained area in the images depicts the hepatic collagen fibrous band.

Prior to the in vivo study, we confirmed that all the rats from each group would have almost the same hepatic fibrosis. Masson staining was used to evaluate the extent of liver fibrosis (Figure 1). We did not observe any differences in the development of fibrosis between the groups. Liver specimens exhibited severe fibrosis, broad and intact septa, marked septation, and rounded contours and were scored 4 using the Laennec fibrosis scoring system, indicating severe fibrosis or early cirrhosis.

**Figure 2:**
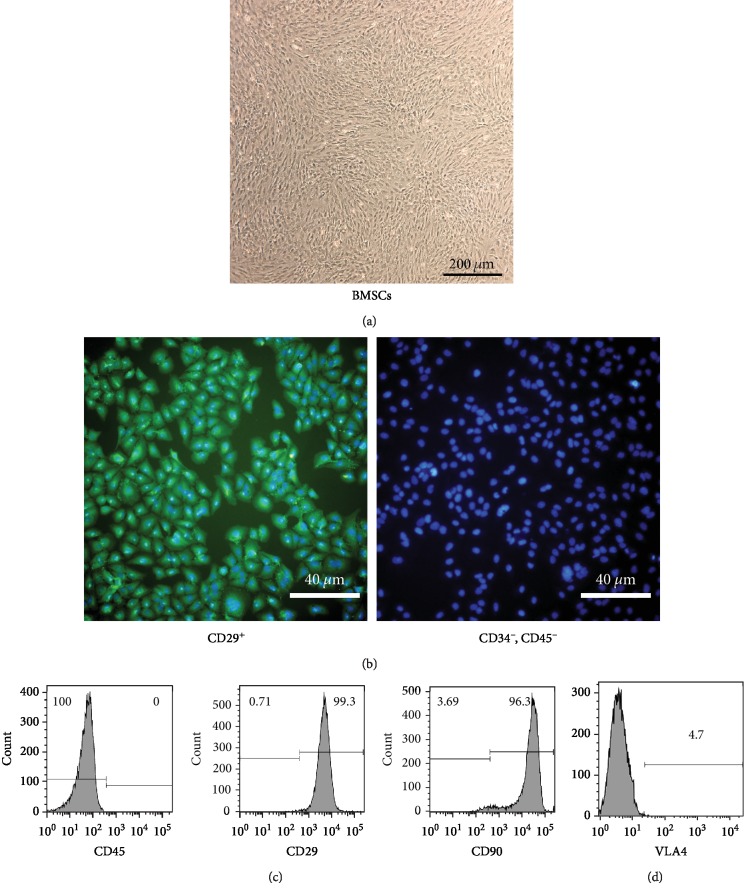
Morphology and identification of BMSCs. (a) Morphology of the BMSCs. Phenotypic characterization of the cultured BMSCs. (b) Immunofluorescent staining of BMSC surface markers CD29, CD34^−^, and CD45^−^. (c) Flow cytometry analysis of the cell surface markers CD45, CD29, and CD90 on cultured BMSCs at the third passage. (d) Flow cytometry analysis of VLA4 expression on cultured BMSCs at the third passage.

The cultured BMSCs needed to be qualified throughout several methods before being transplanted into liver fibrosis model rats. The third passage of BMSCs at the logarithmic phase was collected and examined under a phase-contrast light microscope (CKX31, Olympus, Shinjuku-ku, Tokyo, Japan). The cells were regularly arranged in a swirling pattern and were mostly in long spindle shapes (Figure 2(a)). Immunofluorescent staining revealed that the third passage of BMSCs was CD29-positive and CD34- and CD45-negative (Figure 2(b)). To further characterize BMSCs, cell surface marker proteins were examined by flow cytometric analysis and immunofluorescent staining. Outcomes of the flow cytometry identified high levels of surface antigen CD29 (99.3%) and CD90 (96.3%) and negative of the surface antigen CD45 (0%) (Figure 2(c)). Studies show that VLA4 is mainly expressed on the surface of stem cells and it can bind to VCAM-1 [17]. We confirmed that VLA4 expressed on cultured BMSCs at the third passage by flow cytometry analysis (Figure 2(d)).

**Figure 3:**
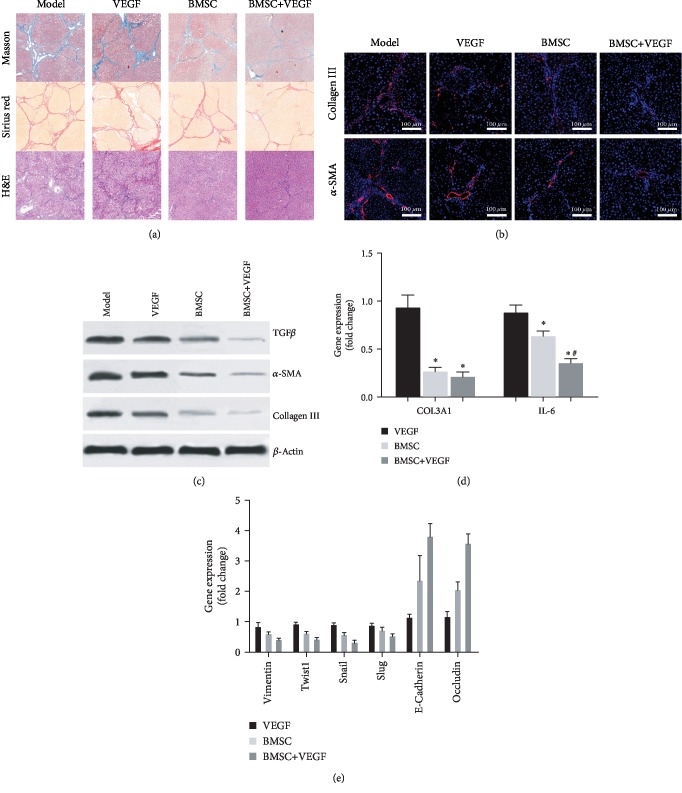
Assessment of liver fibrosis in rats. (a) Three different pathological sections with staining of the liver papillary lobe in four groups (Masson's trichrome stain, Sirius red stain, and H&E stain, *n* = 3/group; original magnification, 40x). (b) Evaluation of the distribution of *α*-smooth muscle actin (*α*-SMA; red) and collagen III (red) in four groups examined by immunofluorescent staining. Nuclei were stained with 4′,6-diamidino-2-phenylindole (DAPI; blue). Scale bars, 100 *μ*m. (c) Protein levels of liver fibrotic markers: TGF*β* (transforming growth factor beta), *α*-SMA, and collagen III in four groups of rats examined by Western blot with *β*-actin used as an internal control. (d) RT-qPCR was used to compare the expression of COL3A1 (collagen type III alpha 1) and IL-6 (interleukin 6) in four groups. (e) EMT-related parameters (vimentin, Twist1, Snail, Slug, E-cadherin, and occludin) were evaluated by RT-qPCR, and GADPH was used as a housekeeping gene. Results are shown as fold changes compared to the model group. ^∗^*p* < 0.05 compared to the model group; ^#^*p* < 0.05 between the BMSC+VEGF group and the BMSC group, *n* = 3.

The extent of liver fibrosis in four groups of rats treated with BMSC and/or VEGF was comprehensively evaluated using tissue staining, immunofluorescent staining, Western blot, and RT-qPCR (Figure 3). The histological sections were scored using the Laennec fibrosis scoring system. Specifically, we identified that the model group exhibited severe fibrosis, broad and intact septa, marked septation, and rounded contours (score 4). The BMSC group exhibited moderately thin septa and incomplete cirrhosis (score 3), while the BMSC+VEGF group exhibited thin and incomplete septa with portal fibrosis (score 2). Finally, the VEGF group exhibited a similar phenotype as the model group (Figure 3(a)). Additionally, immunofluorescent staining revealed that collagen III and *α*-SMA, the indicators responding to liver fibrosis, were distributed in the septa and hepatic parenchyma and specifically enriched in the septa. The model group and the VEGF group exhibited significant collagen deposition, but there was no difference between these two groups. The BMSC group had moderate collagen deposition, with collagen III distributing mainly in the septa and *α*-SMA distributing mainly in the portal area and septa, while the BMSC+VEGF group had the lowest amount of collagen deposition (Figure 3(b)). TGF*β* is an important factor in the formation of liver fibrosis [22]. Protein analysis by Western blot further validated that the BMSC and BMSC+VEGF groups had lower levels of TGF*β* compared to the model and VEGF groups, and the BMSC+VEGF group had the lowest level of TGF*β* expression among all groups (Figure 3(c)). All these results suggest that the expression of profibrotic factors was effectively inhibited in the BMSC+VEGF group.

Importantly, IL-6 (interleukin 6) has also been shown to contribute to the initiation of hepatic fibrosis. Compared to the model group, the BMSC and BMSC+VEGF groups had lower IL-6 mRNA levels, with the lowest IL-6 expression in the BMSC+VEGF group (Figure 3(d)). The VEGF group and the model group had comparable levels of IL-6 and COL3A1 mRNA. Moreover, we also examined the epithelial-mesenchymal transition (EMT) factors at the gene level. Compared to the model group, the BMSC group and the BMSC+VEGF group had decreased expression of vimentin, Twist1, Snail1, and Slug and increased expression of E-cadherin and occludin (Figure 3(e)). Overall, we observed that the gene expression profile in the VEGF group was virtually identical to that of the model group, and the gene expression profiles of the BMSC+VEGF and BMSC groups were also compared.

**Figure 4:**
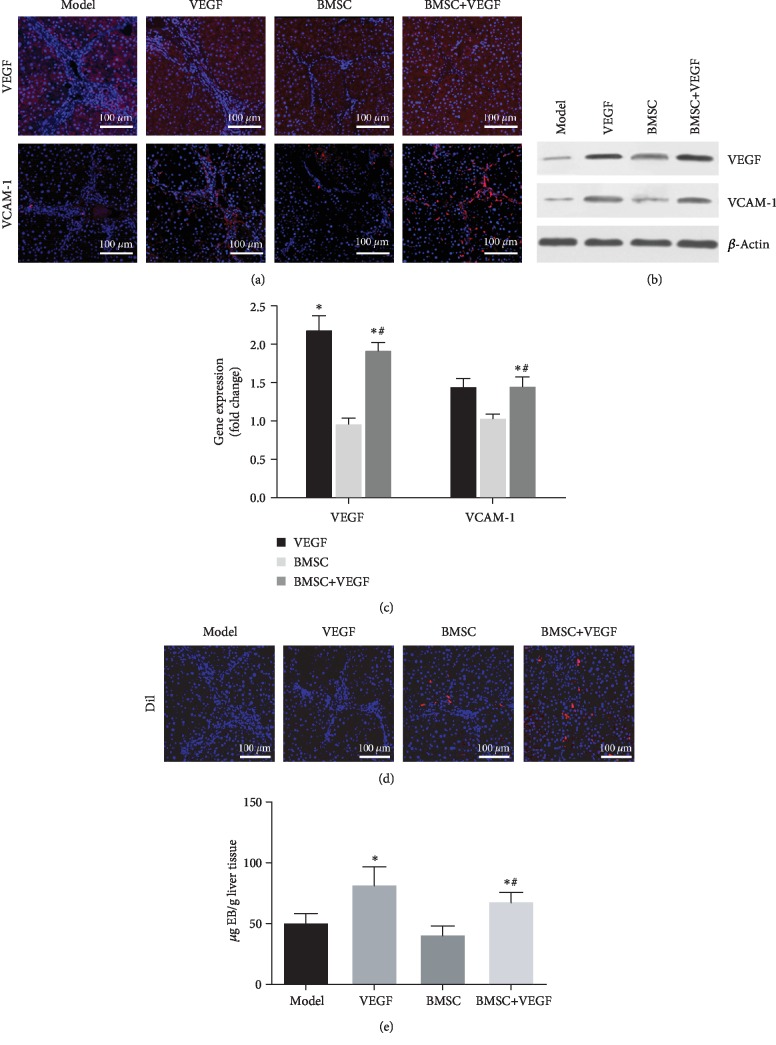
The impact of VEGF on rat liver fibrosis after BMSC transplant. (a) Immunofluorescent staining of VEGF and VCAM-1 (both in red) with nuclei shown in blue. (b) VEGF and VCAM-1 expression in the liver was evaluated by Western blot with *β*-actin used as an internal reference. (c) VEGF and VCAM-1 expression in the liver was evaluated by RT-qPCR, and GADPH was used as a housekeeping gene. Results are shown as fold changes compared to the model group. (d) Immunofluorescent findings 28 days after transplantation of BMSC. DiI-labeled BMSC engraftment in the liver (in red). (e) Residual amount of Evan's blue dye in the liver reflecting hepatic sinusoidal endothelium permeability. ^∗^*p* < 0.05 compared to the model group; ^#^*p* < 0.05 between the BMSC+VEGF group and the BMSC group, *n* = 3.

Furthermore, we examined the VEGF expression levels in the liver using immunofluorescent staining (Figure 4) and observed that the VEGF group and the BMSC+VEGF group had more intense staining compared to the model group (Figure 4(a)), which was recapitulated by VEGF Western blot results (Figure 4(b)). Moreover, RT-qPCR analysis indicated that the VEGF group and the BMSC+VEGF group had significantly higher levels of VEGF mRNA compared to the model group. Moreover, the level of VEGF mRNA in the BMSC+VEGF group was significantly higher compared to that in the BMSC group (Figure 4(c)). Additionally, the levels and pattern of VCAM-1 expression were similar to those of VEGF in the four groups, suggesting elevated VCAM-1 expression in the hepatic sinusoidal endothelial cells (Figures 4(a)–4(c)).

BMSCs were labeled with CM-DiI *in vitro*, and the remaining CM-DiI-labeled cells in the liver were indicative of the engraftment of the donor BMSCs (Figure 4(d)). The BMSC group exhibited a low level of BMSC engraftment, with the cells mainly distributing to the portal and the adjacent septa. In contrast, the BMSC engraftment was higher in the BMSC+VEGF group where the cells were mainly distributed in the liver parenchyma (Figure 4(d)). In addition, we examined the permeability of the hepatic sinusoidal endothelium in the four groups. The retention of Evan's blue in the liver was reflective of the permeability of the hepatic sinusoidal endothelium. Data shown in Figure 4(e) indicate that the VEGF group and the BMSC+VEGF group had the highest level of the residual Evan's blue dye (Figure 4(e)).

**Figure 5:**
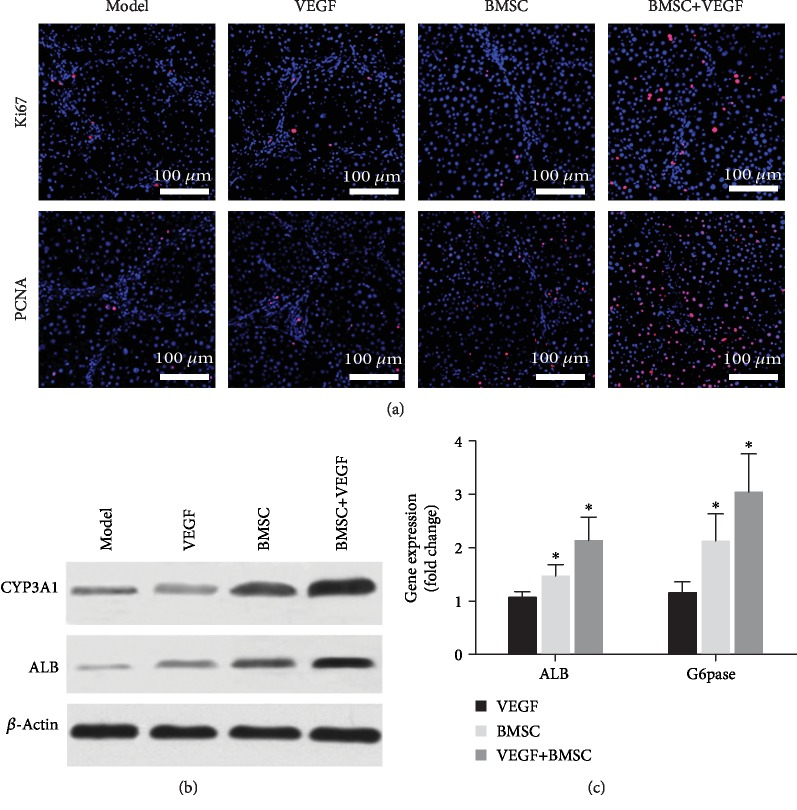
Assessment of liver function. (a) Immunofluorescent staining of Ki67 and PCNA (both in red), with nuclei shown in blue. (b) The expression of CYP3A1 and the expression of ALB in the liver was evaluated by Western blot, and *β*-actin was used as an internal reference. (c) ALB and G6pase expression in the liver was evaluated by RT-qPCR, and GADPH was used as a housekeeping gene. Results are shown as fold changes compared to the model group. ^∗^*p* < 0.05 when compared to the model group, *n* = 3.

To assess the liver function in all four groups of the treated rats, we examined the hepatic expression of Ki67, a nuclear antigen for cell proliferation and PCNA, which is indicative of liver regeneration (Figure 5). Compared to the model group, the BMSC and the BMSC+VEGF group had a higher percentage of Ki67^+^ and PCNA^+^ cells. In addition, compared to the BMSC group, the BMSC+VEGF group had more Ki67^+^ and PCNA^+^ cells, while the VEGF group and the model group had roughly comparable number of proliferating cells (Figure 5(a)).

Moreover, we examined the protein levels of two liver function indicators, CYP3A1 and ALB, by Western blot (Figure 5(b)). Compared to the model group, the BMSC and the BMSC+VEGF group had higher levels of CYP3A1 and ALB. Importantly, levels of CYP3A1 and ALB in the BMSC+VEGF group were higher compared to those in the BMSC group (Figure 5(b)). Furthermore, the mRNA levels of glucose 6-phosphatase (G6pase) and ALB were also examined by RT-qPCR (Figure 5(c)). G6Pase is an important rate-limiting enzyme that regulates hepatic gluconeogenesis [23]. Compared to the model group, the BMSC and the BMSC+VEGF group had higher levels of G6pase and ALB, while the VEGF and model groups were not different. Although G6pase and ALB were slightly higher in the BMSC+VEGF group compared to the BMSC group; the differences were not statistically significant (Figure 5(c)).

**Figure 6:**
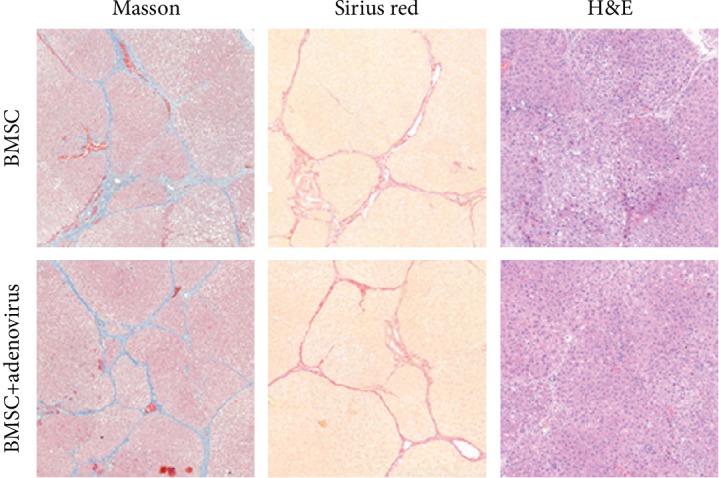
Immunostaining of the BMSC group and the BMSC+adenovirus vector group. Representative histopathological changes observed in Masson's trichrome-, Sirius red-, and H&E-stained liver tissues in the BMSC group and the BMSC+adenovirus vector group; original magnification, 40x.

In order to determine whether adenovirus plays a role in liver fibrosis, two group liver fibrosis rats were treated with BMSCs alone and BMSC+adenoviral vector. From the result above, we found no differences in the level of fibrosis. Both groups had septa of moderate thickness and incomplete fibrosis (Laennec score of 3) (Figure 6).

## 4. Discussion

In this study, we have demonstrated that BMSCs have the ability to alleviate liver fibrosis, including the recovery of markers associated with improved liver function, inhibition of inflammation, and increased hepatocyte regeneration. Moreover, our studies suggest that with the administration of exogenous VEGF, the therapeutic effect of BMSCs on liver fibrosis can be improved.

Many recent reports have been focusing on stem cell therapies to treat end-stage liver diseases [24, 25]. Importantly, many clinical studies have demonstrated that stem cell transplantation improves liver function in patients with liver diseases [26] . However, it has important meaning to know how to increase the engraftment of exogenous stem cells in the injured liver to further improve the efficacy of the therapy. The liver sinusoid, receiving blood from both the portal vein and the hepatic artery, is an important component of the hepatic circulation system. Compared to the other components in the circulation system, the liver sinusoid can be distinguished by two unique features: (1) the hepatic sinusoidal endothelium is fenestrated with specific channels (pore size 6–9 *μ*m) without basal lamina, making it highly permeable; (2) slow blood flow in the sinusoids allows other circulating cells to remain in the liver. These unique features allow circulating cells to migrate into the hepatic parenchyma [2, 27]. Indeed, interaction and migration through the hepatic sinusoidal endothelium is the first step for the circulating stem cell to engraft into the hepatic parenchyma [28, 29]. Consistent with previous studies, histopathological assessment of collagen distribution in the liver of the treated rats confirmed that exogenous BMSC transplant improved liver fibrosis to some extent [3, 30], while VEGF treatment had little effect on liver function (Figure 3). Furthermore, compared to the BMSC transplant only, the combination of VEGF treatment with BMSC transplant significantly reduced hepatic fibrosis in the rat (Figure 3). The predominant cells responsible for liver fibrosis are hepatic stellate cells (HSCs). Studies indicate that TGF*β*1 activates HSCs and is considered as the most potent cytokine that perpetuates fibrotic response in the liver [31]. The BMSC+VEGF group had decreased collagen deposition, reduced stellate cell activation, and decreased TGF*β* and IL-6 expression (inflammation marker), which further suggests that combination of VEGF treatment and BMSC transplant drastically reduces liver fibrosis. Our results suggest that VEGF enhances liver repair mediated by exogenous BMSCs, which likely occurs by promoting BMSC engraftment into the liver.

Many studies have shown that VEGF increases the permeability of the sinusoidal endothelium by binding to VEGFR-1 (*flt-1*) and VEGFR-2 (*KDR/flk-1*), two receptors expressed on endothelial cells [14]. During the progression of liver fibrosis, this mechanism enables the migration of circulating monocytes and macrophages into the liver parenchyma through the sinusoidal endothelium [17]. Our results also showed that VEGF increased sinusoidal endothelial permeability (Figure 4). These results suggest that VEGF promotes exogenous BMSC engraftment by increasing sinusoidal endothelium permeability. Different from the test results of IF and WB, the gene expression of VEGF was slightly decreased; we found that there was no significant statistical difference, and the difference may not have decisive influence on the experimental results (Figure 4(c)). We considered that this may be related to the process by which BMSCs restore the liver. Some of the complex mechanisms affected the posttranscriptional gene expression of VEGF. Meanwhile, we also found that compared with the control group, Evan's blue results showed that the hepatic sinusoidal permeability was slightly decreased after transplantation of BMSCs, maybe caused by VEGF decreasing. (Figure 4(d)). Moreover, through binding to VEGFR-1 (*flt-1*) and VEGFR-2 (*KDR/flk-1*) and activating phospholipase-Cg and NF-*κ*B, VEGF can induce intercellular adhesion molecule-1 (ICAM-1), VCAM-1, and E-selectin in the sinusoidal endothelial cells [18]. The VLA4 on the hematopoietic stem cell mediates hematopoietic stem cell adhesion by interacting with endothelial VCAM-1, its counterreceptor. This interaction facilitates the hematopoietic stem cell attachment to the sinusoidal endothelium and migration into the parenchyma through the sinusoidal endothelium [17]. According to our results, the combination of VCAM-1 and VLA4 may also be a mechanism of promoting BMSC engraftment in the fibrotic liver, although there are only 4.7% positive staining of VLA4. Further research is needed to explore the role of VLA4 in promoting BMSC engraftment. Previous studies demonstrated that the engraftment of stem cells in the liver improves liver function by reducing inflammation and improving the hepatic microenvironment [30, 32]. Therefore, we also examined the effect of exogenous BMSC engraftment on hepatic function. Compared to the BMSC group, the BMSC+VEGF group had higher levels of ALB, CYP, and G6Pase, suggesting that improved BMSC engraftment increases the secretory function of the fibrotic liver. Importantly, the VEGF group did not exhibit significant changes in the liver function compared to the model group. Examination of liver regeneration in our study indicated that PCNA and Ki67 were highly induced in the BMSC+VEGF group but were not different in the VEGF group and the model group. Our data also suggest that VEGF not only promotes BMSC engraftment in the liver but also improves the BMSC-mediated hepatocyte regeneration and liver function.

EMT is one of the indispensable mechanisms for tissue development and during disease progression. In the EMT process, epithelial cells lose their epithelial phenotypes and acquire mesenchymal phenotypes [33]. EMT is an important mechanism during liver fibrosis, by which hepatic cells and bile duct epithelial cells turn into fibroblasts. The hepatic stellate cells also go through the EMT process, become activated and converted into fibroblasts, and are the primary cells contributing to the deposition of the extracellular matrix (e.g., collagen) and fibrosis in the liver [34–36]. Vimentin, Twist1, Snail, and Slug are critical transcription factors (protransforming factors) that are induced in the EMT process [37–39]. E-Cadherin and occludin are also key EMT factors (antitransforming factors), and their expression is suppressed during the EMT process [36]. Based on the results in Figure 3(e), the expression of protransforming factors was decreased and the expression of antitransforming factors was increased post-BMSC transplant, particularly in the BMSC+VEGF group. Overall, these results suggest that the EMT process was inhibited by BMSC transplantation into the liver, which can likely provide one mechanism by which exogenous BMSCs improve hepatic function in liver fibrosis. In addition, the combination of BMSC transplantation with VEGF treatment more effectively inhibited the EMT process, which likely results from VEGF enhancing BMSC engraftment.

## 5. Conclusions

VEGF promotes the engraftment of BMSCs in liver fibrosis, enhances liver regeneration, and improves liver function. The mechanisms maybe related to the increasing hepatic sinusoidal endothelium permeability and VCAM-1-increased expression.

## Figures and Tables

**Table 1:** PCR.

Genes	Reverse primer sequences (5′-3′)	bp
GAPDH	TTCCTACCCCCAATGTATCCG	281
	CATGAGGTCCACCACCCTGTT	
VEGF	GTTCAGAGCGGAGAAAGCATT	83
	CTTGCAACGCGAGTCTGTGT	
COL3A1	GTCGGAGGAATGGGTGGCTAT	322
	CATTGCGTCCATCAAAGCCTC	
G6pase	TGACTATTACAGCAACAGCTCCG	208
	CCAGTATCCCAACCACAAGACG	
ALB	AAATTGGCAACAGACCTCACC	180
	CTCAGCGAGACACTGGGATTT	
E-Cadherin	CCATCGCCTACACCATCCTCA	278
	GGCACCGACCTCATTCTCAAG	
Occludin	CCTGTCTATGCTCGTCATCGTG	125
	CGCTGCCGTAAGGGTAGTTC	
Vimentin	TGACCGCTTCGCCAACTACA	262
	TTCCTCCCTCTGGAGCATCTC	
Twist1	CTACGCCTTCTCCGTCTGGA	252
	TTTAAAAGTGTGCCCCACGC	
Slug	CTGTGACAAGGAATATGTGAGCC	236
	GGTATTTCTTTACATCAGAGTGGG	
Snail	AGTTCACCTTCCAGCAGCCCTA	214
	CTTTTGCCACTGTCCTCATCG	
IL-6	AGGATACCACCCACAACAGACC	109
	TTGCCATTGCACAACTCTTTTC	
VCAM-1	TGAACCCAAACAAAGGCAGAGTA	147
	TTGGGAGTTGGAAAACCATCAC	

**Table 2:** Laennec scoring system for staging fibrosis in liver biopsies.

Stage	Name	Septa (thickness and number)	Criteria	Score
0	No definite fibrosis			0
1	Minimal fibrosis	+/−	No septa or rare thin septum; may have portal expansion or mild sinusoidal fibrosis	1
2	Mild fibrosis	+	Occasional thin septa; may have portal expansion or mild sinusoidal fibrosis	2
3	Moderate fibrosis	++	Moderate thin septa; up to incomplete cirrhosis	3
4A	Cirrhosis, mild, definite, or probable	+++	Marked septation with rounded contours or visible nodules. Most septa are thin (one broad septum allowed)	4
4B	Moderate cirrhosis	++++	At least two broad septa, but no very broad septa and less than half of biopsy length composed of minute nodules	5
4C	Severe cirrhosis	+++++	At least very broad septum or more than half of biopsy length composed of minute nodules	6

## Data Availability

The datasets generated during and/or analyzed during the current study are available from the corresponding author on reasonable request.

## Abbreviations

MSC: Mesenchymal stem cell
VEGF: Vascular endothelial growth factor
VCAM-1: Vascular adhesion molecule-1
BMSC: Bone marrow mesenchymal stem cell
CCl_4_: Carbon tetrachloride
CCM: Complete culture medium
FBS: Fetal bovine serum
H&E Staining: Hematoxylin-eosin staining
IF: Immunofluorescent
*α*-SMA: Alpha-smooth muscle actin
PCNA: Proliferating cell nuclear antigen
TGF*β*: Transforming growth factor beta
ALB: Albumin
RT-qPCR: Quantitative reverse transcription polymerase chain reaction
VLA4: Very late antigen-4
EMT: Epithelial-mesenchymal transition
G6pase: Glucose 6-phosphatase
HSC: Hepatic stellate cell
IL-6: Interleukin 6.
